# Novel compound heterozygous mutations in *SCN4A* as a potential genetic cause contributing to myopathic manifestations: A case report and literature review

**DOI:** 10.1016/j.heliyon.2024.e28684

**Published:** 2024-03-22

**Authors:** Ji Yoon Han, Joonhong Park

**Affiliations:** a Department of Pediatrics, College of Medicine, The Catholic University of Korea, Seoul, 06591, Republic of Korea; b Department of Laboratory Medicine, Jeonbuk National University Medical School and Hospital, Jeonju, 54907, Republic of Korea; c Research Institute of Clinical Medicine of Jeonbuk National University-Biomedical Research Institute of Jeonbuk National University Hospital, Jeonju, 54907, Republic of Korea

**Keywords:** Voltage-gated sodium channel alpha subunit 4, *SCN4A*, Congenital myopathy, Targeted exome sequencing

## Abstract

**Background:**

*SCN4A* mutations account for a diverse array of clinical manifestations, encompassing periodic paralysis, myotonia, and newly recognized symptoms like classical congenital myopathy or congenital myasthenic syndromes. We describe the initial occurrence of myopathic features mimic with recessive classical CM in a Korean infant presenting with novel compound heterozygous *SCN4A* mutations. The infant exhibited profound hypotonia after birth, thereby expanding the spectrum of *SCN4A*-related channelopathy.

**Methods:**

The genetic analyses comprised targeted exome sequencing, employing a Celemics G-Mendeliome DES Panel, along with Sanger sequencing.

**Results:**

Considering the clinical manifestations observed in the proband, *SCN4A* variants emerged as the primary contenders for autosomal recessive (AR) congenital myopathy 22a, classic (#620351). Sanger sequencing validated the association of *SCN4A* variants with the phenotype, affirming the AR nature of the compound heterozygous variants in both the carrier mother (c.3533G > T/p.Gly1178Val) and the father (c.4216G > A/p.Ala1406Thr).

**Conclusion:**

Our report emphasizes the association of *novel* compound heterozygous mutations in *SCN4A* with myopathic features resembling CM, as supporting by muscle biopsy. It is essential to note that pathogenic *SCN4A* LoF mutations are exceedingly rare. This study contributes to our understanding of *SCN4A* mutations and their role in myopathic features mimic with classical CM.

## 1 Introduction

The *SCN4A* gene, which codes for the alpha subunit of the skeletal muscle voltage-gated sodium channel (NaV1.4), is situated on chromosome 17q23-25. NaV1.4 assumes a crucial role in muscle-related disorders by governing the initiation and transmission of action potentials [1]. The voltage-gated sodium channel consists of a solitary pore-forming alpha subunit and two separate beta subunits [2]. The alpha subunit, a singular polypeptide chain, undergoes folding into four homologous yet non-identical repeats (repeats I to IV). Each repeat encompasses six transmembrane segments (S1 to S6) [3], S1 to S4 constitute the voltage-sensing domain, while S5-6 form the pore domain. The positively charged S4 segments function as voltage sensors, regulating the opening and closing of the pore. Upon insertion into the membrane, these four repeats create a central pore with segments five and six lining its walls, while S4 serves as the channel's voltage sensor owing to its positively charged amino acids. The loop (P loop) between S5 and S6 in the extracellular domain determines ion selectivity. NaV1.4 rapidly activates (<1 msec) in response to depolarization, allowing a substantial inward Na + current (∼5 mA/cm2), leading to the immediate generation of action potentials (∼500 mV/msec). Rapid inactivation shortly after the action potential's generation prevents repetitive discharges, ensuring normal skeletal muscle contraction and maintaining the physiological excitability of the sarcolemma [2].

Mutations in the *SCN4A* gene exert a significant influence on skeletal muscle excitability, impacting the inactivation or activation of muscle ion channels [2]. These mutations give rise to a diverse range of clinical manifestations, ranging from periodic paralysis (PP) to myotonia. Recently, they have been linked to classical congenital myopathy (CM, #620351), severe fetal hypokinesia, congenital myasthenic syndromes (CMS, #614198), exercise intolerance, myalgia, sudden infant death syndrome, and severe neonatal episodic laryngospasms. Mutations in *SCN4A* can disrupt muscle hyper-excitability by altering channel kinetics or functions. However, discerning the specific contributions of fast and slow inactivation to the phenotype can be challenging [4]. The influence of abnormal slow inactivation has been noted in non-dystrophic myotonia and periodic paralysis (PP) [5]. The gradual activation of NaV1.4 assumes a crucial role in regulating muscle contraction, particularly in fast-twitch muscles characterized by elevated levels of muscle firing [6]. Gain-of-function (GoF) *SCN4A* mutations elevate the levels of inward Na + current, resulting in conditions such as hyperkalemic periodic paralysis (hyperPP, OMIM #170500), hypokalemic periodic paralysis (hypoPP, #613345), myotonia congenita (MC, #608390), and paramyotonia congenita (PMC, #168300) with autosomal dominant (AD) inheritance [7]. Conversely, loss-of-function (LoF) *SCN4A* mutations are less common and are linked to autosomal recessive (AR) diseases [2]. These mutations contribute to congenital myasthenic syndromes (CMS), which are characterized by muscle weakness stemming from impaired transmission of electrical signals at the neuromuscular junction, or hypoPP with AD inheritance [4,8]. Recently, classical CM has been reported in pediatric patients from various ethnic backgrounds, harboring either homozygous or compound heterozygous mutations of *SCN4A* with AR inheritance [[9], [10], [11], [12], [13]].

To date, various small genetic studies and case series have been conducted on Korean patients diagnosed with hypoPP, hyperPP, MC, or PMC, all attributed to mono-allelic *SCN4A* mutations [[14], [15], [16], [17], [18], [19], [20], [21], [22], [23], [24], [25], [26], [27]]. In this report, we describe the initial occurrence of myopathic features mimic with recessive classical CM in a Korean infant presenting with novel compound heterozygous *SCN4A* mutations. The infant exhibited profound hypotonia after birth, thereby expanding the spectrum of *SCN4A*-related channelopathy.

## 2 Case presentation

**Fig. 1:**
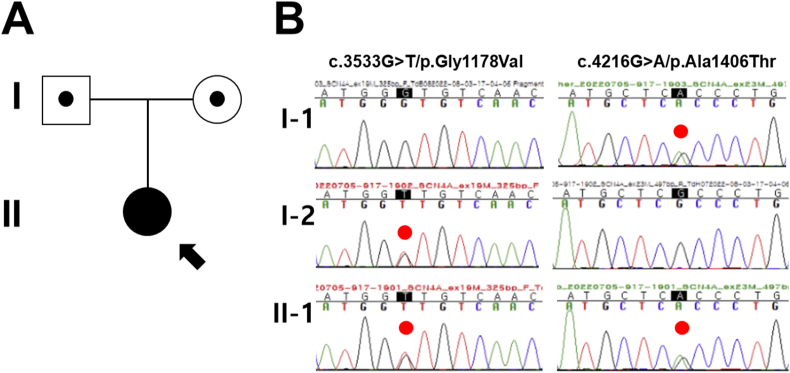
Pedigree and segregation analysis. (A) Pedigree of the proband (arrow) carrying compound heterozygous *SCN4A* variants in an autosomal recessive (AR) inheritance and her family members. (B) Sanger sequencing confirmed bi-allelic nonsynonymous *SCN4A* variants, c.3533G > T/p.Gly1178Val and c.4216G > A/p.Ala1406Thr of AR origin in the proband (II-1) (Reference transcript ID: NM_000334.4). Asymptomatic parents of the proband are obligate heterozygotes. Each *SCN4A* variant is emphasized in the red dot. (For interpretation of the references to colour in this figure legend, the reader is referred to the Web version of this article.)

The female proband (II-1 in Fig. 1A) was delivered by cesarean section at 37 weeks of gestational age to non-consanguineous parents. Prenatal complications included polyhydramnios and fetal hypokinesia. The parents had no known history of neuromuscular disorders. At birth, her weight was 2200 g (7th percentile), height was 48 cm (10-25th percentile), head circumference was 33 cm (10-25th percentile), and her 1-min APGAR score was 8. She required 3 minutes of positive pressure ventilation and received heated humidified high-flow nasal cannula treatment for two weeks in the neonatal intensive care unit. The patient faced challenges with swallowing, leading to G-tube feeding for three months and intravenous nutrition for one month. Facial features included an elongated face, blepharophimosis, and a high-arched palate. Developmental reflexes, including Moro, grasp, and suckling reflexes, were weak. Special feeding methods were employed due to muscle weakness and the high-arched palate. Importantly, there was no evidence of myotonia, fluctuating muscle weakness, tongue fasciculations, limitations in eyeball movements, or heightened deep tendon reflexes. Early motor development was significantly delayed, but she eventually gained head control at four months and sat independently at six months. Echocardiography indicated normal ejection fractions and the absence of structural abnormalities. At five months, single-fiber electromyography (EMG) showed variation in the action potential time interval in almost all muscle fibers, but nerve conduction was normal. A left biceps muscle biopsy revealed mild variation in size and degeneration and regeneration of a few myofibers. Skeletal radiology at six months showed no abnormalities. By seven months, her overall development was appropriate for her age, except for motor growth. Laboratory investigations, including tests for creatine kinase (CK), thyroid function, lactate, and pyruvate, all fell within normal ranges. The patient also tested negative for anti-Acetylcholine receptor antibody. Brain magnetic resonance images displayed a myelination pattern appropriate for her age without structural abnormalities, and visual and auditory provoked potential tests yielded normal results.

## 3 Genetic testing

**Fig. 2:**
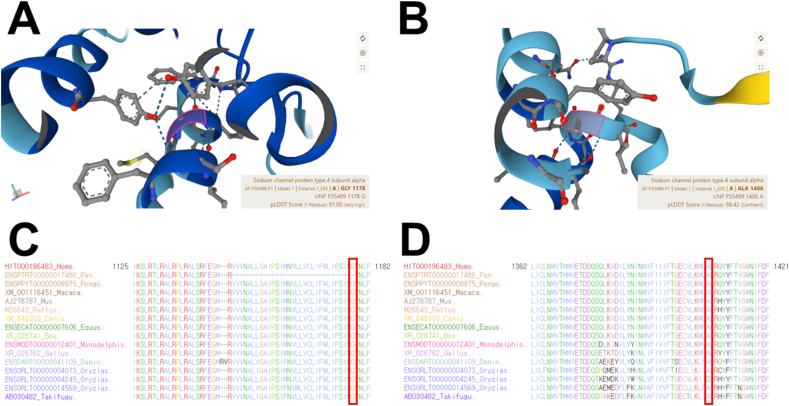
Protein structure analysis and conservation of the SCN4A codon change in the proband. (A and B) Protein structure analysis using AlphaFold showed very high per-residue confidence score (pLDDT) of 91.00 for p.Gly1178Val (A, pLDDT >90) and a confident score of 88.42 for p.Ala1406Thr (B, 70 < pLDDT <90) of the SCN4A protein. Each protein residue is highlighted in pink box. (C and D) Sequence alignment of the conserved fifth transmembrane segment in the third domain (S5DIII) and the loop between second and third transmembrane segment in the fourth domain (loop S2DIV-S3DIV) of NaV1.4 channel in multiple vertebrate species. Both the Glycine in position 1178 (C) and Alanine residues in position 1406 (D) are highly conserved across all vertebrate species (highlighted in the red empty box). (For interpretation of the references to colour in this figure legend, the reader is referred to the Web version of this article.)

In light of the congenital hypotonia, developmental delay, and myopathic facial dysmorphisms observed, we pursued sequential genetic testing for various neuromuscular disorders. These included spinal muscular atrophy types I, II, and III, myotonic dystrophy type I, and myotonic dystrophy type II. Unfortunately, no pathogenic mutations were identified in these initial tests. Subsequently, we employed targeted exome sequencing utilizing the Celemics G-Mendeliome Clinical Exome Sequencing Panel, which covers 5870 genes associated with inherited Mendelian disorders. Massively parallel sequencing was carried out with the DNBSEQ-G400RS High-throughput Sequencing Set and DNBSEQ-G400 sequencer. The interpretation of pathogenic variants followed the standards and guidelines set by the American College of Medical Genetics and Genomics (ACMG) and the Association for Molecular Pathology (AMP) [28]. Ultimately, the analysis revealed compound heterozygous variants in two genes: *SCN4A* (c.3533G > T/p.Gly1178Val and c.4216G > A/p.Ala1406Thr) and *SYNE1* (c.1904G > A/p.Trp635* and c.24832+3A > G). The clinical presentation of the proband was consistent with *SCN4A* variants, indicative of AR CM 22a, classic (#620351). Sanger sequencing confirmed the segregation of *SCN4A* variants with the phenotype and established the AR status of the compound heterozygous variants in both the carrier mother (c.3533G > T/p.Gly1178Val) and father (c.4216G > A/p.Ala1406Thr) (Fig. 1B). All in-silico analyses predicted that these *SCN4A* variants are likely pathogenic. Furthermore, we conducted an analysis of the SCN4A protein structure for amino acid stability using AlphaFold [29]. When analyzing the SCN4A protein structure for amino acid stability using AlphaFold, very high and confident per-residue confidence scores (pLDDT) of 91.00 and 88.42 were calculated for SCN4A p.Gly1178 (Fig. 2A) and p.Ala1406 (Fig. 2B) residues, as highlighted in pink box, respectively. In addition, a sequence alignment of the conserved cytoplasmic domain of the SCN4A protein in multiple vertebrate species revealed high conservation of the protein sequence of the SCN4A p.Gly1178 (Fig. 2C) and p.Ala1406 (Fig. 2D) residues across all multiple vertebrate species, as emphasized in the red empty box, respectively. Notably, the c.3533G > T/p.Gly1178Val variant was not found in dbSNP, gnomAD, or KRGDB, classifying it as a novel variant.

## 4 Discussion

CM represent a rare and genetically diverse category of neuromuscular disorders, with an estimated prevalence as indicated by meta-analysis studies, ranging from 0.52 to 50.1 per 100,000 in the pediatric population [29]. These conditions typically manifest with symptoms emerging in the neonatal period. Among these cases, 60%–80% are linked to central nervous system (CNS) involvement, while 12%–34% have a peripheral etiology. Among patients who remain undiagnosed, 82% exhibit symptoms suggestive of muscle diseases [[30], [31], [32]]. Notably, the onset of symptoms occurs in the neonatal period, affecting approximately 14% of patients with neonatal hypotonia [33]. Muscle weakness in CM patients is typically characterized as static or slowly progressive, often with normal CK levels. In these conditions, the CNS remains unaffected. Although severe hypotonia may be present in early infancy, it generally stabilizes over time or shows improvement with age. Traditionally, neuromuscular diseases were classified based on histopathological features. However, with the identification of causative genes, the classification has evolved to emphasize the involvement of specific genes. Disease-causing genes have been identified in 50%–75% of CM cases, encompassing over 40 associated genes, including *ACTA1*, *DNM2*, *MTM1*, *NEB, RYR1,* and *SEPN1* [34].

**Fig. 3:**
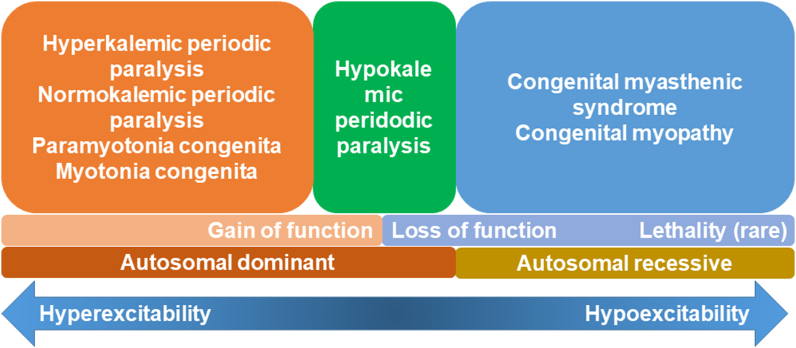
The clinical spectrum and functional effect of *SCN4A*-related skeletal muscle channelopathy.

Nav1.4 is the pore-forming subunit of the main sodium channel present in skeletal muscles and related channelopathies that affect skeletal muscle excitability are associated with muscular dysfunctions. In this case, compound heterozygous *SCN4A* variants can cause abnormal Nav1.4 activity and result in myophatic features including hypotonia, muscle weakness and myopathic morphologies. Both *SCN4A* variants are suspected to be like pathogenic according to in-silico prediction, analysis of the SCN4A structures, and conservation scores [35]. Efforts to discern various entities within *SCN4A*-related skeletal muscle channelopathies have focused on pathophysiological analysis, clinical manifestations, and genetic findings. However, similar mutations can lead to comparable effects on membrane hypo- or hyperactivity, and a definitive distinction between GoF and LoF based on *SCN4A* variants is not fully established [36]. Over the past decade, cellular electrophysiology studies have played a pivotal role in uncovering *SCN4A* mutations, particularly LoF mutations in NaV1.4, which have been causally linked to muscle weakness in conditions such as hypoPP, CMS, and CM [37] (Fig. 3). Among familial hypoPP cases, missense mutations in *SCN4A* are more prevalent and result in sustained membrane depolarization [4,36]. Studies involving mice with bi-allelic null mutations of *SCN4A* have demonstrated that NaV1.5 does not compensate for NaV1.4 in skeletal muscle at birth, underscoring the critical role of NaV1.4 [9,38]. Utilizing a knockout mouse model, it was shown that an acute reduction in Na + current was necessary to induce myasthenia [38]. The recessively inherited myasthenia or CM conditions suggest a gene-dosage effect and are associated with mutant alleles exhibiting partial LoF or null mutations [39].

**Fig. 4:**
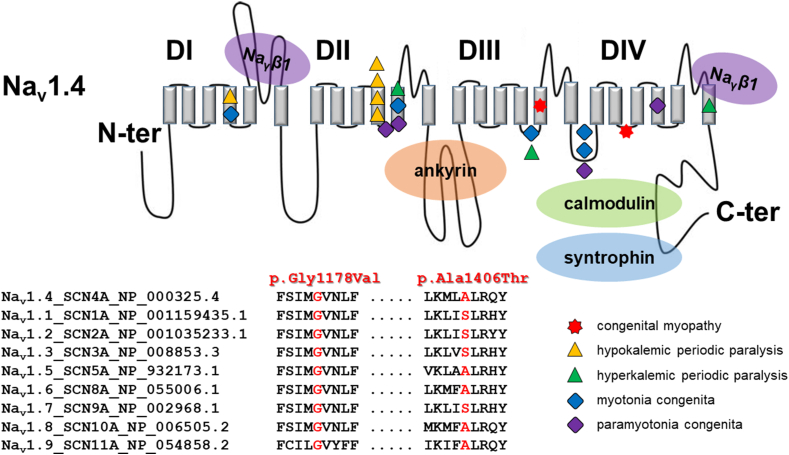
Location of the 16 different *SCN4A* (likely) pathogenic variants reported in the Korean patients diagnosed with *SCN4A*-associated diseases on the secondary structure of NaV1.4 channel. The *SCN4A* variants are indicated by a red star for congenital myopathy, a yellow triangle for hypokalemic periodic paralysis, a green triangle for hyperkalemic periodic paralysis, a blue rhombus for myotonia congenita, and a violet rhombus for paramyotonia congenita. The position of all variants has been established using NextProt (https://www.nextprot.org/entry/NX_P35499/sequence). The locations of the p.Gly1178Val and p.Ala1406Thr are shown by a red star in S5DIII and the loop S2DIV-S3DIV, respectively, as illustrated at the bottom of the figure, along with an alignment against all human Nav isoforms (National Center for Biotechnology Information identifiers provided in parentheses). The location of the p.Gly1178Val and p.Ala1406Thr is shown in red; the p.Gly1178Val is conserved across all human Na_v_ channels, however, the p.Ala1406Thr is not conserved. (For interpretation of the references to colour in this figure legend, the reader is referred to the Web version of this article.)

In 2015, *SCN4A* was initially associated with an AR inheritance pattern and CMS [8]. Subsequently, several cases of CMS or CM associated with LoF variants were reported [9,10,12]. A total of 18 variants, including missense, splicing, nonsense, and frameshift mutations, were identified in 12 probands from 12 families across six studies. These variants segregated with CM or CMS in an additional 14 family members [[8], [9], [10],^12^,^13^,^40^,^41^] (Table 1). Many patients with these conditions exhibited decreased fetal movements and maternal polyhydramnios. After birth, they presented with a spectrum of muscle weakness, ranging from mild generalized hypotonia to severe weakness that posed survival challenges. Similar to CM, most patients, including our case, showed improvements in motor function over time, with a resolution of respiratory and feeding difficulties. In our case, the variant p.3533.G > T (p.G1178V) is a *novel* variant, although there are reports that a mutation (c.3539A > T, p.N1180I) in a similar locus has caused myotonia or myopathy [42,43]. Cross-species comparisons of SCN4A protein sequences indicated high conservation of these mutated regions in vertebrates. Glycine 1178 is conserved across all human voltage-gated sodium channel genes (NaV1.1-NaV1.9), while Alanine 1406 is consistently conserved as either Alanine or Serine across all human Nav channels (Fig. 4). In patients with CMS, a homozygous mutation (c.4252A, p.I1418V) of *SCN4A* was confirmed, and this locus is near our variant (c.4216G > A, p.Ala1406Thr) [44]. These findings provide additional support for the pathogenicity of the proband's variants. Mutations in the *SYNE1* gene in the proband can give rise to a range of conditions, including Emery-Dreifuss muscular dystrophy (AD inheritance), Arthrogryposis multiplex congenita (AR inheritance), or Spinocerebellar ataxia (AR inheritance). Interestingly, each mutation has been inherited from asymptomatic parents, and clinical manifestations do not appear to be correlated across these different conditions.

**Table 1:** *SCN4A* mutations in congenital myasthenic syndrome/congenital myopathy patients.

References (year)	SCN4A mutation	Amino acid change	Type of mutations	Domain	Functional consequence	Reported in channelopathy	Muscle biopsy	EMG/NCS	Electrophysiologic studies	Remarks
**Berghold et al. (2022)**	c.4360C > T c.3615C > G	p.R1454W p.N1205K	Missense Missense	IV III	LoF NA	HyperPP	Normal finding	Normal/normal	NA	Initial diagnosis: mitochondrial disease, parents: asymptomatic carrier
**Elia et al. (2019)**	c.4378C > T	p.R1460W (homozygous)	Missense	IV	LoF		NA	No myotonic discharges/NA	Reduced amplitude and hyperpolarized shift of inactivation	Parents: asymptomatic carrier
	c.4378C > T c.3175C > T	p.R1460W p.R1059X	Missense Nonsense	IV IV	LoF NA		Myopathy	Myotonic discharges	NA	
**Gonorazky et al. (2017)**	c.3245G > A c.1123T > C	p.A1142Q p.C375A	Missense Missense	IV III	LoF LoF		Myopathy	NA/NA	decrement of the action potential and subsequent reduction of muscle contraction	Myopathy, MRI: muscle atrophy, parents: asymptomatic carrier
**Mercier et al. (2017)**	c.3359C > T c.1036+1G > A	p.S1120L p.?	Missense Splicing	III III	NA NA		Myopathic and dystrophic features	Myopathic pattern/normal		Parents: asymptomatic carrier
**Habbout et al. (2015)**	c.4360C > T (homozygous)	p.R1454W	Missense	IV	LoF		NA	NA	Decremental muscle response after a long exercise	Results of EMG was normal at resting states
**Zaharieva et al. (2015)**	c.311G > A c.3403C > T	p.R104H p.R1135C	Missense Missense	I IV	LoF NA	HypoPP	Myopathy	Normal/NA	Reduced channel activity	Family 1, MRI: muscle atrophy
	c.673C > T c.3626G > T	p.R225W p.C1209F	Missense Missense	I III	LoF LoF	Myotonia	Myopathy	Myopathic change without myotonia/NA	Reduced channel activity	Family 2, MRI: muscle atrophy
	c.1480C > T c.5345dup	p.Q470X p.H1782Qfs65	Nonsense Frameshift	I C-Terminus	NA LoF		Myopathy	NA/NA	NA	Family 3
	c.3205G > A c.3145-2A > C	p.D1069 N p.A1049VfsX50	Missense Splicing	III III	LoF NA	hyperPP	Myopathy	Myopathic change without myotonia/NA	Reduced channel activity	Family 4
	c.1144C > A (homozygous)	p.P382T	Missense	I	LoF NA		Myopathy	NA/NA	Reduced channel activity	Family 5
	c.608T > A c.4779C > A	p.M203K p.Y1593X	Missense Nonsense	I IV	LoF NA	hyperPP	Myopathy	NA/NA	Reduced channel activity	Family 6
**Our case**	c.3533G > T c.4216G > A	p.G1178V p.A1406T	Missense Missense	III IV	NA NA	HyperPP	Myopathy	Normal	NA	Parents: asymptomatic carrier

LoF: loss of function, GoF: gain of function, NA: not available, EMG: electromyography, NCS: nerve conduction velocity, MRI: magnetic resonance imaging.

The genotype-phenotype correlations in *SCN4A*-related myotonia and periodic paralyses remain unclear, as the clinical phenotype in Korean patients carrying *SCN4A* mutations did not seem to be affected by the mutation's location in the NaV1.4 channel (Fig. 4). LoF of the NaV1.4 channel resulted in reduced action potential and subsequent muscle contraction. In older individuals with hyperPP, some develop chronic progressive myopathy with constant weakness [45]. Reports on AD periodic paralyses and myotonia associated with heterozygous *SCN4A* mutations in Korean patients have been limited in the literature, typically involving small cohorts and focusing on single clinical subgroups. This report presents the first case of a Korean patient exhibiting recessive myopathic features that mimic CM and provides an overview of molecular and clinical features in a moderate-sized cohort of Korean patients with *SCN4A*-related channelopathies, based on an extensive literature review. Genotype-phenotype correlations from studies on Korean patients with *SCN4A* mutations are summarized in Table 2. Our patient showed generalized hypotonia after birth and a clinical improvement during early infancy. EMG testing revealed normal response and muscle biopsy results suggest CM rather than CMS. Treatment with acetazolamide and salbutamol showed limited efficacy, while pyridostigmine therapy was associated with side effects when administered at suboptimal doses to patients with CM and/or CMS caused by *SCN4A* variants [13]. Our patient also experienced adverse effects, such as nausea and hypersalivation, with acetazolamide. Membrane activity dysfunction has resulted in various myopathic features, and the distinction between GoF and LoF is not clearly defined. We propose that compound heterozygous SCN4A variants can give rise to myopathic features resembling CM, and a more accurate nomenclature would be 'SCN4A-related skeletal muscle channelopathy.

**Table 2:** Studies reporting genotype-phenotype correlations in Korean patients carrying *SCN4A* mutations.

Phenotype	S/A	Base change	Codon change	Location in NaV1.4 channel	Inheritance	References
CM	F/7 m	c.3533G > T	p.Gly1178Val	S5DIII	AR	Our case
CM	F/7 m	c.4216G > A	p.Ala1406Thr	loop S2DIV-S3DIV	AR	Our case
HypoPP	M/16	c.664C > T	p.Arg222Trp	S4DI	AD	Park et al. (2010)
MC	M/11y	c.673C > T	p.Arg225Trp	S4DI	AD	Lee et al. (2009)
HypoPP	M/17y	c.2006G > A	p.Arg669His	S4DII	AD	Kim et al. (2007)
HypoPP	M/28y	c.2006G > A	p.Arg669His	S4DII	AD	Kim et al. (2021)
HypoPP	M/15y	c.2015G > A	p.Arg672His	S4DII	AD	Kim et al. (2011)
HypoPP	M/12y	c.2014C > T	p.Arg672Cys	S4DII	AD	Kim et al. (2007)
HypoPP	M/15y	c.2014C > T	p.Arg672Cys	S4DII	AD	Kim et al. (2004)
HypoPP	M/6y	c.2014C > G	p.Arg672Gly	S4DII	AD	Kim et al. (2007)
HypoPP	F/6y	c.2014C > G	p.Arg672Gly	S4DII	AD	Kim et al. (2007)
HypoPP	M/8y	c.2014C > G	p.Arg672Gly	S4DII	AD	Kim et al. (2007)
PMC	M/5y	c.2078T > C	p.Ile693Thr	loop S4DII-S5DII	AD	Lee et al. (2009)
HyperPP	F/16y	c.2111C > T	p.Thr704Met	S5DII	AD	Han et al. (2011)
HyperPP	M/49	c.2111C > T	p.Thr704Met	S5DII	AD	Lee et al. (2015)
HyperPP	M/47	c.2111C > T	p.Thr704Met	S5DII	AD	Lee et al. (2015)
HyperPP	M/45	c.2111C > T	p.Thr704Met	S5DII	AD	Lee et al. (2015)
HyperPP + MC	M/51y	c.2111C > T	p.Thr704Met	S5DII	AD	Jeong et al. (2018)
HyperPP + MC	M/49y	c.2111C > T	p.Thr704Met	S5DII	AD	Jeong et al. (2018)
HyperPP + MC	M/47y	c.2111C > T	p.Thr704Met	S5DII	AD	Jeong et al. (2018)
HyperPP + PMC	M/9y	c.2111C > T	p.Thr704Met	S5DII	AD	Kim et al. (2001)
HyperPP + PMC	M/7y	c.2111C > T	p.Thr704Met	S5DII	AD	Kim et al. (2001)
MC	M/30y	c.3466G > A	p.Ala1156Thr	loop S4DIII-S5DIII	AD	Lee et al. (2009)
HyperPP	M/27y	c.3466G > A	p.Ala1156Thr	loop S4DIII-S5DIII	AD	Lee et al. (2009)
MC	M/19y	c.3877G > A	p.Val1293Ile	loop DIII-DIV	AD	Chung et al. (2016)
MC	M/1 m	c.3917G > A	p.Gly1306Glu	loop DIII-DIV	AD	Lee et al. (2009)
MC	M/32y	c.3917G > A	p.Gly1306Glu	loop DIII-DIV	AD	Lee et al. (2009)
PMC	F/24y	c.3917G > T	p.Gly1306Val	loop DIII-DIV	AD	Park et al. (2010)
PMC	M/33y	c.4342C > T	p.Arg1448Cys	S4DIV	AD	Kim et al. (2002)
HyperPP	M/10y	c.4774A > G	p.Met1592Val	S6DIV	AD	Lee et al. (2010)
HyperPP	F/8y	c.4774A > G	p.Met1592Val	S6DIV	AD	Lee et al. (2010)
HyperPP	F/6y	c.4774A > G	p.Met1592Val	S6DIV	AD	Lee et al. (2010)

The location of SCN4A mutations in NaV1.4 channel was established using NextProt (https://www.nextprot.org/entry/NX_P35499/sequence).

S/A, sex/age; S, transmembrane segment; D, domain (or repeat); loop, region intracellular/extracellular between 2 segments and 2 domain; CM, congenital myopathy; HypoPP, hypokalemic periodic paralysis; MC, myotonia congenita; PMC, paramyotonia congenita; HyperPP, hyperkalemic periodic paralysis; AR; autosomal recessive; AD, autosomal dominant.

In conclusion, the clinical phenotype and electrophysiological studies reveal significant overlap between CM and CMS, especially in young infants [46,47]. Consequently, the diagnosis of *SCN4A*-related skeletal muscle channelopathy may be more appropriate than attempting to classify it as either CMS or CM. Our report emphasizes the association of *novel* compound heterozygous mutations in *SCN4A* with myopathic features resembling CM, as supporting by muscle biopsy. It is essential to note that pathogenic *SCN4A* LoF mutations are exceedingly rare. This study contributes to our understanding of *SCN4A* mutations and their role in myopathic features mimic with classical CM. Nonetheless, further research is imperative to unveil the mechanisms behind CMS/CM resulting from *SCN4A* mutations, including genotype-phenotype correlations and underlying mechanisms, before we can consider the possibility of gene-based therapies.

## Ethics approval and consent to participate

This study was approved by the Institutional Review Board (IRB) of the Catholic University of Korea (Approval number: DC23ZASI0003; Date of approval: January 17, 2023).

## Consent for publication

Written informed consent was obtained from the parents on behalf of their child for the clinical and molecular analyses and for the publication of any potentially identifiable images or data included in this study.

## Data availability statement

Clinical and genetic data of this patient were described in this article.

## Funding

The authors wish to acknowledge the financial support of the Catholic Medical Center Research Foundation made in the program year of 2022. This work was supported by a grant from the 10.13039/501100003725National Research Foundation of Korea (NRF), funded by the Korean government (Ministry of Science and ICT (10.13039/501100014188MSIT): 2021R1F1A1063568). This article was supported by research funds for newly appointed professors of 10.13039/501100015499Jeonbuk National University in 2020. This paper was supported by Fund of Biomedical Research Institute, 10.13039/501100015499Jeonbuk National University Hospital.

## CRediT authorship contribution statement

**Ji Yoon Han:** Writing – review & editing, Writing – original draft, Resources, Data curation. **Joonhong Park:** Writing – review & editing, Writing – original draft, Visualization, Validation, Methodology, Formal analysis.

## Declaration of competing interest

The authors declare that they have no known competing financial interests or personal relationships that could have appeared to influence the work reported in this paper.
